# Enabling Efficient Scheduling of Multi-Type Sources in Power Systems via Uncertainty Monitoring and Nonlinear Constraint Processing

**DOI:** 10.3390/s25216564

**Published:** 2025-10-24

**Authors:** Di Zhang, Qionglin Li, Ji Han, Chunsun Tian, Yebin Li

**Affiliations:** 1 Electric Power Research Institute of State Grid Henan Electric Power Company, Zhengzhou 450000, China; 2 College of New Energy, Harbin Institute of Technology at Weihai, Weihai 264200, China

**Keywords:** uncertainty monitoring, nonlinear constraint processing, optimization-based scheduling, renewable energy integration, hydropower modeling, multi-source coordination

## Abstract

The large-scale integration of renewable energy sources introduces significant uncertainty into modern power systems, posing new challenges for reliable and economical operation. Effective scheduling therefore requires accurate monitoring of uncertainty and efficient handling of nonlinear system dynamics. This paper proposes an optimization-based scheduling method that combines sensor-informed monitoring of photovoltaic (PV) uncertainty with advanced processing of nonlinear hydropower characteristics. A detailed hydropower model is incorporated into the framework to represent water balance, reservoir dynamics, and head–discharge–power relationships with improved accuracy. Nonlinear constraints and uncertainty are addressed through a unified approximation scheme that ensures computational tractability. Case studies on the modified IEEE −39 system show that the proposed method achieves effective multi-source coordination, reduces operating costs by up to 2.9%, and enhances renewable energy utilization across different uncertainty levels and PV penetration scenarios.

## 1. Introduction

The rapid transition toward carbon neutrality and the large-scale integration of renewable energy sources are reshaping the structure and operation of modern power systems. Variable wind and photovoltaic (PV) generation introduce strong volatility and uncertainty, significantly increasing the requirements for system flexibility, reliability, and economic performance. At the same time, conventional thermal power and hydropower remain indispensable for balancing supply and demand. Consequently, developing optimization-based scheduling methods that can coordinate multiple heterogeneous resources under uncertainty and nonlinearity has become a critical research direction.

Optimization-based scheduling has been widely studied as an effective approach to coordinate multi-type power sources and address the challenges of system flexibility, renewable integration, and operational efficiency. References [1,2] establish coordinated planning frameworks that exploit demand response and nodal carbon potential to jointly optimize economic and environmental objectives. In [3], the flexible retrofitting of thermal power units combined with energy storage deployment is investigated, where the benefits of peak shaving and renewable accommodation are quantitatively evaluated. Reference [4] introduces a source–load cooperative peak-shaving mechanism that reduces curtailment and improves the efficiency of renewable energy utilization. Reference [5] proposes an improved day-ahead scheduling strategy that incorporates DC frequency support to enhance renewable accommodation and peak-shaving performance in interconnected grids. Building on day-ahead models, Reference [6] develops a combined day-ahead and intraday framework, where rolling optimization is used to reduce fluctuations and improve stability. Reference [7] further introduces a real-time scheduling method based on dynamic decision-making, enabling continuous adjustment of system operating points to address unpredictable power flow deviations.

With the growing share of renewable energy, the variability and intermittency of wind and PV generation have introduced significant uncertainty into power system scheduling. Such uncertainty directly affects the balance of supply and demand, as well as system security and economic efficiency. Consequently, uncertainty modeling has become an essential component of modern scheduling research. Distributionally robust and data-driven scheduling approaches have been applied to improve system resilience against renewable volatility [8,9]. For instance, robust two-stage dispatch methods have been developed for wind–thermal coordination [10], while chance-constrained formulations have been extended to integrated electricity–gas systems [11]. Further, interval optimization [12] and probabilistic admissibility assessment [13] have been proposed to balance modeling fidelity and tractability. These methods demonstrate the importance of explicitly addressing uncertainty, yet they often rely on simplified linear models that may not adequately capture the nonlinear dynamics of hydropower and other flexible units. This creates a critical research gap, i.e., while uncertainty-aware methods have advanced considerably in modeling renewable variability, the flexible resources used to balance this variability—particularly hydropower—are typically represented through aggregated or linearized approximations that ignore essential physical constraints. Such simplifications may lead to suboptimal or infeasible scheduling solutions when detailed operational requirements must be satisfied in practice.

To cope with renewable uncertainty, the regulation capability of hydropower has been gradually incorporated into dispatch models. Owing to its fast response and large storage capacity, hydropower can provide both short-term balancing and long-term flexibility, making it an indispensable resource for enhancing system stability and accommodating variable renewable energy. Recent research has extensively examined the role of hydropower in complementary scheduling frameworks. Reference [14] develops a short-term scheduling model for the hydropower station and evaluates its performance under multiple operating scenarios. In [15], a bi-objective optimization model is constructed to maximize hydropower generation while simultaneously smoothing output fluctuations. Reference [16] investigates two scheduling strategies, one minimizing total output volatility and the other enhancing overall stability. Adaptive operating rules that link short- and long-term scheduling horizons are proposed in [17], highlighting the need to balance hydropower flexibility with renewable variability. To address the stochastic nature of renewable generation, Reference [18] applies a probabilistic collocation method based on polynomial chaos expansion, thereby supporting the secure operation of cascade hydropower systems. Reference [19] embeds short-term fluctuation risks into a mid- to long-term optimization framework, improving the robustness of hydropower scheduling decisions. Furthermore, Reference [20] emphasizes water-use efficiency by formulating a short-term cascade hydropower optimization model with the objective of minimizing water consumption.

Although hydropower has been increasingly incorporated into scheduling models, most existing studies treat its operation in a simplified manner, often using linear approximations or fixed efficiency factors. In reality, hydropower scheduling is characterized by multiple nonlinear relationships, such as the water level–storage curve, the tail water level–discharge function, and the nonlinear mapping between water head, discharge, and power output. Additional complexities arise from operating constraints, including vibration zone avoidance, output fluctuation limits, and head loss effects, all of which directly influence the feasible operating space. Ignoring or oversimplifying these nonlinear characteristics not only reduces the accuracy of hydropower representation but may also lead to suboptimal or even infeasible scheduling outcomes in practical applications. Therefore, there exists a significant gap between uncertainty-aware scheduling frameworks and detailed physical modeling of flexible resources, hindering the development of truly practical and reliable multi-source coordination strategies.

In recent years, considerable efforts have been devoted to addressing the nonlinear nature of scheduling models, and a variety of approximation and reformulation techniques have been proposed [21,22]. Frequency-related nonlinear indices such as nadir and RoCoF are commonly embedded into dispatch through piecewise linearization, which transforms inherently nonlinear expressions into tractable mixed-integer linear forms [23,24]. To cope with complex nonlinear coupling between renewable generation and system inertia, synthetic inertia from inverter-based resources has been modeled with linearized approximations [25]. Decomposition methods have also been developed to separate nonlinear multi-area or multi-timescale problems into smaller subproblems, improving tractability while retaining the essential nonlinear relationships [26]. Convex relaxation techniques have been applied to replace nonconvex constraints with equivalent convex envelopes, thereby accelerating the solution of large-scale nonlinear scheduling problems [27]. In addition, unit commitment formulations considering nonlinear dynamic responses have been approximated through bi-level or linearized models [28], and multi-area frequency-constrained scheduling has been reformulated with linear surrogates to handle otherwise intractable nonlinear dynamics.

However, these linearized methods cannot be directly applied to hydropower scheduling, since its nonlinearities stem from water level–storage and tail water level–discharge relationships, as well as the bilinear dependence of head, discharge, and power output. Additional constraints such as vibration zones and head loss make the problem more complex, requiring dedicated modeling approaches beyond conventional linearization or relaxation techniques.

To address these challenges, this paper proposes an optimization-based scheduling method that coordinates hydropower, thermal power, and PV generation under uncertainty and nonlinearity. The main contributions are as follows:

1. A detailed hydropower model is established, incorporating water balance, reservoir dynamics, operational constraints, and nonlinear head–discharge–power characteristics to ensure physical feasibility and scheduling accuracy.
2. A linearization framework is proposed to approximate nonlinear hydropower constraints, including reservoir storage, tail water level–discharge relations, vibration zones, head loss, and turbine power characteristics, enabling accurate representation of physical features while maintaining computational tractability. The proposed nonlinear constraint processing framework extends beyond traditional convex relaxation and static piecewise linearization by introducing adaptive, physically interpretable reformulations for hydropower-related nonlinearities. Through tailored linearization of head loss, vibration zone, and turbine power coupling constraints, the method maintains global model linearity while dynamically balancing computational efficiency and accuracy.
3. A comprehensive scheduling framework is constructed that simultaneously accounts for uncertainty and nonlinearity, enabling more realistic and reliable multi-source coordination.

The remainder of this paper is organized as follows. Section 2 formulates the optimization-based scheduling model for coordinating multi-type power sources. Section 3 introduces the proposed methods for handling nonlinearity and uncertainty. Section 4 discusses the case study and result analysis. Finally, Section 5 concludes the paper.

## 2. Scheduling Model for Multi-Type Sources in Power Systems

### 2.1. Objective Functions

To achieve coordinated optimization of thermal power, hydropower, and PV generation, this paper constructs a multi-objective scheduling function that comprehensively considers thermal power operating costs, water curtailment costs, and PV curtailment costs, as follows:

(1) $$\left\{\begin{array}{l} F_{1}=\sum_{t=1}^{T}\sum_{m=1}^{M}\left(a_{G,m}\cdot u_{m,t}^{\mathrm{fire}}\cdot P_{m,t}^{\mathrm{fire}}+a_{G,m}\cdot u_{m,t}^{\mathrm{fire}}\right)\\ F_{2}=\sum_{t=1}^{T}\sum_{i=1}^{I}\left(P_{i,t}^{\mathrm{hydro},f}-P_{i,t}^{\mathrm{hydro}}\right)\\ F_{3}=\sum_{t=1}^{T}\sum_{j=1}^{J}\left({\tilde{P}}_{j,t}^{\mathrm{pv},f}-P_{j,t}^{\mathrm{pv}}\right) \end{array}\right.$$

(2) $$F=w_{1}\cdot F_{1}+w_{2}\cdot F_{2}+w_{3}\cdot F_{3}$$

where $F_{1}$ represents the thermal power unit operating cost [29]; $F_{2}$ represents the water curtailment cost; $F_{3}$ represents the PV curtailment cost; $u_{m,t}^{\mathrm{fire}}$ is the on/off status variable of the *m*-th thermal power unit in time period t, where $u_{m,t}^{\mathrm{fire}}\in \left\{0,1\right\}$, taking 1 indicates the on state and 0 indicates the off state; $P_{i,t}^{\mathrm{hydro}}$ is the output of hydropower i in time period t; $P_{j,t}^{\mathrm{pv}}$ is the output of PV j in time period t; $P_{m,t}^{\mathrm{fire}}$ is the output of thermal power m in time period t; $P_{i,t}^{\mathrm{hydro},f}$ is the predicted output value of hydropower i in time period t; ${\tilde{P}}_{j,t}^{\mathrm{pv},f}$ is the predicted output value of PV j in time period t, which are uncertain quantities; I, J, M represent hydropower, PV, and thermal power in the power system, respectively; T is the total number of scheduling periods in the power system; $w_{1}$, $w_{2}$, $w_{3}$ are objective function weights.

### 2.2. Constraints

#### 2.2.1. Thermal Power Operating Constraints

To ensure reasonable and safe system operation, thermal power units must satisfy output constraints, start/stop state change constraints, start/stop time constraints, ramping constraints, and reserve constraints [30]:

(3) $$u_{m,t}^{\mathrm{fire}}\cdot P_{\min,m}^{\mathrm{fire}\;}\leq P_{m,t}^{\mathrm{fire}\;}\leq u_{m,t}^{\mathrm{fire}}\cdot P_{\max,m}^{\mathrm{fire}\;}$$

(4) $$u_{m,t}-u_{m,t-1}=y_{m,t}^{\mathrm{on}}-y_{m,t}^{\mathrm{off}}$$

(5) $$y_{m,t}^{\mathrm{on}}+y_{m,t}^{\mathrm{off}}\leq 1$$

(6) $$\begin{array}{l} \sum_{\tau =t}^{t+FT_{\mathrm{on},m}^{\mathrm{fire}}-1}u_{m,\tau }^{\mathrm{fire}}\geq FT_{\mathrm{on},m}^{\mathrm{fire}}\cdot y_{m,t}^{\mathrm{on},\mathrm{fire}},\;t\leq T-FT_{\mathrm{on},m}^{\mathrm{fire}}+1\\ \sum_{\tau =t}^{T}u_{m,\tau }^{\mathrm{fire}}\geq (T-t+1)\cdot y_{m,t}^{\mathrm{on},\mathrm{fire}},\;t>T-FT_{\mathrm{on},m}^{\mathrm{fire}}+1 \end{array}$$

(7) $$\left\{\begin{array}{l} \sum_{\tau =t}^{t+FT_{\mathrm{off},m}^{\mathrm{fire}}-1}u_{m,\tau }^{\mathrm{fire}}\geq FT_{\mathrm{off},m}^{\mathrm{fire}}\cdot y_{m,t}^{\mathrm{off},\mathrm{fire}},\;t\leq T-FT_{\mathrm{off},m}^{\mathrm{fire}}+1\\ \sum_{\tau =t}^{T}u_{m,\tau }^{\mathrm{fire}}\geq (T-t+1)\cdot y_{m,t}^{\mathrm{off},\mathrm{fire}},\;t>T-FT_{\mathrm{off},m}^{\mathrm{fire}}+1 \end{array}\right.$$

(8) $$P_{m,t+1}^{\mathrm{fire}}-P_{m,t}^{\mathrm{fire}}\leq V_{m}^{\mathrm{up}}$$

(9) $$P_{m,t}^{\mathrm{fire}}-P_{m,t+1}^{\mathrm{fire}}\leq V_{m}^{\mathrm{down}}$$

(10) $$\left\{\begin{array}{l} P_{m,t}^{\mathrm{fire}}+R_{m,t}^{\mathrm{fire}+}\leq u_{m,t}^{\mathrm{fire}}\cdot P_{\max,m}^{\mathrm{fire}}\\ P_{m,t}^{\mathrm{fire}}-R_{m,t}^{\mathrm{fire}-}\geq u_{m,t}^{\mathrm{fire}}\cdot P_{\min,m}^{\mathrm{fire}} \end{array}\right.$$

where Equation (3) represents thermal power unit output constraints; Equations (4) and (5) represent thermal power unit start/stop state change constraints; Equations (6) and (7) represent thermal power unit start/stop time constraints; Equations (8) and (9) represent thermal power unit ramping constraints. $P_{\min,m}^{\mathrm{fire}\;}$ is the minimum output of unit m; $P_{\max,m}^{\mathrm{fire}\;}$ is the maximum output of unit m; $y_{m,t}^{\mathrm{on},\mathrm{fire}}$ and $y_{m,t}^{\mathrm{off},\mathrm{fire}}$ are binary variables, where 1 indicates unit m starts up in time period t, and 0 indicates unit m shuts down in time period t; $FT_{\mathrm{on},m}^{\mathrm{fire}}$ is the minimum on-time duration of the *m*-th thermal power unit; $FT_{\mathrm{off},m}^{\mathrm{fire}}$ is the minimum off-time duration of the *m*-th thermal power unit; $V_{m}^{\mathrm{up}}$ is the up-ramping rate of the *m*-th thermal power unit; $V_{m}^{\mathrm{down}}$ is the down-ramping rate of the *m*-th thermal power unit; $R_{m,t}^{\mathrm{fire}+}$ and $R_{m,t}^{\mathrm{fire}-}$ are the positive and negative reserve capacities of the *m*-th thermal power unit.

To improve renewable energy integration levels and prevent thermal power from occupying excessive system load share when renewable energy output is sufficient, this paper sets the following thermal power output constraint to dynamically limit its output upper bound:

(11) $$\sum_{m=1}^{M}P_{m,t}^{\mathrm{fire}}\leq \min\{\sum_{m=1}^{M}P_{m,\max}^{\mathrm{fire}},\beta \times \left(\sum_{i=1}^{I}P_{m,t}^{\mathrm{hydro}}+\sum_{j=1}^{J}P_{j,t}^{\mathrm{pv}}\right)\}$$

where β is the regulation coefficient used to control the response degree of thermal power output to renewable energy (hydropower and PV) output.

#### 2.2.2. Hydropower Operating Constraints

Hydrological resource and reservoir regulation constraints mainly include water balance, water level upper and lower limits, reservoir initial and final water level control, water level–storage relationship, and tail water level–discharge relationship. Water balance constraints ensure that reservoir storage changes in each period satisfy conservation relationships with inflow, outflow, generation, and spillage flows, as shown in Equations (12)–(14); water level upper and lower limit constraints restrict reservoir operating water levels within safe ranges to prevent extreme water levels from affecting dam safety and generation efficiency, as shown in Equation (15); initial and final water level control constraints stipulate that the starting and ending water levels of the scheduling period should be close to set values to ensure continuity and controllability of multi-period scheduling, as shown in Equations (16) and (17); the water level–storage relationship describes the nonlinear mapping between reservoir water level and storage capacity, which is key to achieving hydrological calculations and constraint conversion, as shown in Equation (18); the tail water level–discharge relationship reflects the coupling characteristics between water level and discharge after water flows through the power station, helping achieve upstream and downstream coordinated scheduling and ecological flow control, as shown in Equation (19).

(12) $$V_{i,t}=V_{i,t-1}+3600\left(I_{i,t}-Q_{i,t}\right)\Delta t$$

(13) $$I_{i,t}=Q_{i-1,t-\tau }+R_{i,t}$$

(14) $$Q_{i,t}^{\mathrm{hydro}}=Q_{i,t}^{\mathrm{hydro},p}+Q_{i,t}^{\mathrm{hydro},d}$$

(15) $${\underline{Z}}_{i,t}^{\mathrm{up}}\leq Z_{i,t}^{\mathrm{up}}\leq {\bar{Z}}_{i,t}^{\mathrm{up}}$$

(16) $$Z_{i,1}^{\mathrm{up}\;}=Z_{i,\mathrm{begin}}^{\mathrm{up}\;}$$

(17) $$\left|Z_{i,T}^{\mathrm{up}\;}-Z_{i,\;\mathrm{end}\;}^{\mathrm{up}\;}\right|\leq \Delta Z$$

(18) $$V_{i,t}=f_{i}^{\mathrm{ZV}}\left(Z_{i,t}^{\mathrm{up}}\right)$$

(19) $$Z_{i,t}^{\mathrm{down}\;}=f_{i}^{\mathrm{ZQ}}\left(Q_{i,t}^{\mathrm{hydro}}\right)$$

where $V_{i,t}$ is the storage capacity; $I_{i,t}$ is the inflow; $Q_{i,t}^{\mathrm{hydro}}$ is the outflow; $R_{i,t}$ is the interval flow; τ is the water travel time; $Q_{i,t}^{\mathrm{hydro},p}$ and $Q_{i,t}^{\mathrm{hydro},d}$ are the generation flow and spillage flow of power station i in time period t, respectively; $Z_{i,t}^{\mathrm{up}}$, ${\bar{Z}}_{i,t}^{\mathrm{up}}$, ${\underline{Z}}_{i,t}^{\mathrm{up}}$ are the forebay water level and its upper and lower limits of the reservoir; $Z_{i,\mathrm{begin}}^{\mathrm{up}\;}$ and $Z_{i,\;\mathrm{end}\;}^{\mathrm{up}\;}$ are the initial water level and final control water level of the scheduling period; ΔZ is the allowable final water level deviation of the scheduling period to avoid affecting water quantity in the next scheduling cycle; $f_{i}^{\mathrm{ZV}}\left(\cdot \right)$ is the nonlinear water level–storage relationship curve function of the reservoir where power station i is located; $f_{i}^{\mathrm{ZQ}}\left(\cdot \right)$ is the nonlinear relationship curve function between tail water level and discharge of power station i; $Z_{i,t}^{\mathrm{down}\;}$ is the tail water level of power station i in time period t.

Output and operating status constraints are mainly used to regulate the power output and operating behavior of hydropower units, ensuring equipment safety, smooth operation, and scheduling feasibility. Among them, output limit constraints control the upper and lower output limits of units in each period, ensuring they do not exceed unit capacity ranges, as shown in Equation (20); vibration zone avoidance constraints prevent units from operating in vibration zones for extended periods, reducing mechanical wear and operating risks, as shown in Equation (21); start/stop operation constraints limit minimum start/stop duration and start/stop frequency, preventing efficiency degradation and equipment damage caused by frequent switching, as shown in Equations (22)–(25); ramping capability constraints limit the rate of output change per unit time, ensuring smoothness of output change processes, as shown in Equation (26); output fluctuation limit constraints restrict the duration of increasing and decreasing output, reducing adverse effects of sudden water flow changes on ecology and equipment while improving operating stability, as shown in Equation (27).

(20) $$u_{i,t}^{\mathrm{hydro}}{\underline{P}}_{i,t}^{\mathrm{hydro}}\leq P_{i,t}^{\mathrm{hydro}}\leq u_{i,t}^{\mathrm{hydro}}{\bar{P}}_{i,t}^{\mathrm{hydro}}$$

(21) $$\left(P_{i,t}^{\mathrm{hydro}}-P_{\max,i,k}^{\mathrm{hydro}}\right)\left(P_{i,t}^{\mathrm{hydro}}-P_{\min,i,k}^{\mathrm{hydro}}\right)⩾0$$

(22) $$u_{i,t}^{\mathrm{hydro}\;}-u_{i,t-1}^{\mathrm{hydro}\;}=y_{i,t}^{\mathrm{on},\mathrm{hydro}\;}-y_{i,t}^{\mathrm{off},\mathrm{hydro}\;}$$

(23) $$y_{i,t}^{\mathrm{on},\mathrm{hydro}\;}+\sum_{\lambda =t+1}^{t+T_{\mathrm{on},i}^{\mathrm{hydro}}-1}y_{i,\lambda }^{\mathrm{on},\mathrm{hydro}\;}\leq 1$$

(24) $$y_{i,t}^{\mathrm{off},\mathrm{hydro}\;}+\sum_{\lambda =t+1}^{t+T_{\mathrm{off},i}^{\mathrm{hydro}}-1}y_{i,\lambda }^{\mathrm{off},\mathrm{hydro}\;}\leq 1T_{\mathrm{off},i}^{\mathrm{hydro}}$$

(25) $$\sum_{t=1}^{T}y_{i,t}^{\mathrm{on},\mathrm{hydro}\;}⩽M_{i}^{\mathrm{on}}$$

(26) $$-\Delta P_{i}^{\mathrm{hydro}\;}\leq P_{i,t}^{\mathrm{hydro}\;}-P_{i,t-1}^{\mathrm{hydro}\;}\leq \Delta P_{i}^{\mathrm{hydro}\;}$$

(27) $$\left(P_{i,t}^{\mathrm{hydro}\;}-P_{i,t-\sigma -1}^{\mathrm{hydro}\;}\right)\left(P_{i,t}^{\mathrm{hydro}\;}-P_{i,t-1}^{\mathrm{hydro}\;}\right)⩾0,\sigma =1,2,\cdots ,t_{e}-1$$

where ${\bar{P}}_{i,t}^{\mathrm{hydro}}$ and ${\underline{P}}_{i,t}^{\mathrm{hydro}}$ are the upper and lower output limits of power station i in time period t; $u_{i,t}^{\mathrm{hydro}}$ is the on/off status variable of power station i in time period t, where $u_{i,t}^{\mathrm{hydro}}\in \left\{0,1\right\}$, taking 1 indicates on state and 0 indicates off state; $P_{\max,i,k}^{\mathrm{hydro}}$ and $P_{\min,i,k}^{\mathrm{hydro}}$ are the lower and upper output limits of the k-th vibration zone of power station i; $y_{i,t}^{\mathrm{on},\mathrm{hydro}\;}\in \left\{0,1\right\}$ is the startup operation variable of power station i in time period t, taking 1 indicates startup operation; $y_{i,t}^{\mathrm{off},\mathrm{hydro}\;}\in \left\{0,1\right\}$ is the shutdown operation variable of power station i unit in time period t, taking 1 indicates shutdown operation; $T_{\mathrm{on},i}^{\mathrm{hydro}}$ and $T_{\mathrm{off},i}^{\mathrm{hydro}}$ are the minimum on and off durations of power station, respectively; $M_{i}^{\mathrm{on}}$ is the maximum number of startups of power station i during the scheduling period to avoid frequent start/stop operations; $\Delta P_{i}^{\mathrm{hydro}\;}$ is the ramping capability of power station i; $t_{e}$ is the minimum number of periods that a unit must maintain during one round of output increase/decrease process, where $t_{e}>1$.

Hydraulic and water-energy conversion characteristic constraints are mainly used to characterize the hydraulic and equipment physical characteristics in the water-to-electricity conversion process, ensuring physical feasibility of generation scheduling results. Among them, generation flow constraints stipulate that the water consumption for unit generation must operate within allowable ranges, as shown in Equation (28); generation head relationship constraints combine water level changes and flow conditions to calculate the effective head available for generation, as shown in Equation (29); head loss function expresses the energy loss generated during water flow through intake and discharge structures, as shown in Equation (30); unit dynamic characteristic constraints reflect the coupling relationship between output, head, and flow through nonlinear functions, which is an important basis for accurately evaluating generation capacity and optimizing scheduling decisions, as shown in Equation (31).

(28) $$u_{i,t}^{\mathrm{hydro}\;}{\underline{Q}}_{i}^{\mathrm{hydro}}\leq Q_{i,t}^{\mathrm{hydro}}\leq u_{i,t}^{\mathrm{hydro}\;}{\bar{Q}}_{i}^{\mathrm{hydro}}$$

(29) $$H_{i,t}^{\mathrm{hydro}}=\frac{Z_{i,t}^{\mathrm{up}}+Z_{i,t}^{\mathrm{down}\;}}{2}-H_{i,t}^{\mathrm{hydro},\mathrm{loss}}$$

(30) $$H_{i,t}^{\mathrm{hydro},\mathrm{loss}}=a_{i}{\left(Q_{i,t}^{\mathrm{hydro}}\right)}^{2}+b_{i}$$

(31) $$P_{i,t}^{\mathrm{hydro}}=f_{i}^{\mathrm{NHQ}}\left(Q_{i,t}^{\mathrm{hydro}},H_{i,t}^{\mathrm{hydro}}\right)=\eta_{i}\cdot \rho \cdot g\cdot Q_{i,t}^{\mathrm{hydro}}\cdot H_{i,t}^{\mathrm{hydro}}$$

where $Q_{i,t}^{\mathrm{hydro}}$, ${\bar{Q}}_{i}^{\mathrm{hydro}}$, ${\underline{Q}}_{i}^{\mathrm{hydro}}$ are the generation flow and its upper and lower limits of power station i in time period t; $H_{i,t}^{\mathrm{hydro}}$ and $H_{i,t}^{\mathrm{hydro},\mathrm{loss}}$ are the generation head and head loss of power station i in time period t; $a_{i}$ and $b_{i}$ are the head loss coefficient and loss constant of power station i, which can generally be obtained through hydraulic experiments; $f_{i,n}^{\mathrm{NHQ}}\left(\cdot \right)$ is the nonlinear relationship function of output-head-flow of power station i; $\eta_{i}$ is the comprehensive efficiency of hydropower station i; ρ is the density of water; g is gravitational acceleration.

#### 2.2.3. Other Constraints

PV output must be within the predicted output, and considering PV uncertainty, its operating constraints can be expressed as:

(32) $$\min P_{r}\left\{P_{j,t}^{\mathrm{pv}}\leq {\tilde{P}}_{j,t}^{\mathrm{pv},f}\right\}\geq \alpha$$

where α is the confidence level.

The power system must satisfy power balance constraints:

(33) $$\min P_{r}\left\{\sum_{i=1}^{I}P_{i,t}^{\mathrm{hydro}}+\sum_{j=1}^{J}{\tilde{P}}_{j,t}^{\mathrm{pv}}+\sum_{m=1}^{M}P_{m,t}^{\mathrm{fire}}-\sum_{n=1}^{N}{\tilde{P}}_{n,t}^{\mathrm{load}}=0\right\}\geq \alpha$$

where ${\tilde{P}}_{n,t}^{\mathrm{load}}$ is the load demand at node n in time period t, which is an uncertain quantity; N is the number of load nodes.

Additionally, considering power system reserve constraints:

(34) $$\min P_{r}\left\{\sum_{n=1}^{N}{\tilde{P}}_{n,t}^{\mathrm{load}}-\sum_{j=1}^{J}{\tilde{P}}_{j,t}^{\mathrm{pv}}-\sum_{i=1}^{I}P_{i,\max}^{\mathrm{hydro}}-\sum_{m=1}^{M}P_{m,\max}^{\mathrm{fire}}\leq 0\right\}\geq \alpha$$

## 3. Methods for Handling Nonlinearity and Uncertainty

### 3.1. Nonlinearity in Scheduling Model

The nonlinear constraints in the scheduling model include Equations (18), (19), (21), (27), (30) and (31).

For the water level–storage relationship, i.e., Equation (18), a piecewise linear interpolation function can be employed:

(35) $$V_{i,t}=\left\{\begin{array}{ll} V_{i,1}+\frac{V_{i,2}-V_{i,1}}{Z_{i,2}-Z_{i,1}}\cdot \left(Z_{i,t}^{\mathrm{up}}-Z_{i,1}\right), & Z_{i,1}\leq Z_{i,t}^{\mathrm{up}}<Z_{i,2}\\ V_{i,2}+\frac{V_{i,3}-V_{i,2}}{Z_{i,3}-Z_{i,2}}\cdot \left(Z_{i,t}^{\mathrm{up}}-Z_{i,2}\right), & Z_{i,2}\leq Z_{i,t}^{\mathrm{up}}<Z_{i,3}\\ \vdots & \\ V_{i,n-1}+\frac{V_{i,n}-V_{i,n-1}}{Z_{i,n}-Z_{i,n-1}}\cdot \left(Z_{i,t}^{\mathrm{up}}-Z_{i,n-1}\right), & Z_{i,n-1}\leq Z_{i,t}^{\mathrm{up}}\leq Z_{i,n} \end{array}\right.$$

where $Z_{i,k}$ and $V_{i,k}$ represent the k-th water level point and its corresponding reservoir capacity of reservoir i, respectively; $Z_{i,t}^{\mathrm{up}}$ is the dam water level of reservoir i during period t; $V_{i,t}$ is the corresponding reservoir capacity; the interpolation interval $\left[Z_{i,k},Z_{i,k+1}\right]$ is provided by the reservoir operating curve table; n is the number of known water level–storage data points in the curve table.

The tail water level–discharge relationship, i.e., Equation (19), can similarly be represented using a piecewise linear interpolation function:

(36) $$Z_{i,t}^{\mathrm{down}}=\left\{\begin{array}{ll} Z_{1}+\frac{Z_{2}-Z_{1}}{Q_{2}-Q_{1}}\cdot \left(Q_{i,t}^{\mathrm{hydro}}-Q_{1}\right), & Q_{1}\leq Q_{i,t}^{\mathrm{hydro}}<Q_{2}\\ Z_{2}+\frac{Z_{3}-Z_{2}}{Q_{3}-Q_{2}}\cdot \left(Q_{i,t}^{\mathrm{hydro}}-Q_{2}\right), & Q_{2}\leq Q_{i,t}^{\mathrm{hydro}}<Q_{3}\\ \vdots & \\ Z_{n-1}+\frac{Z_{n}-Z_{n-1}}{Q_{n}-Q_{n-1}}\cdot \left(Q_{i,t}^{\mathrm{hydro}}-Q_{n-1}\right), & Q_{n-1}\leq Q_{i,t}^{\mathrm{hydro}}\leq Q_{n} \end{array}\right.$$

where $\left(Q_{k},Z_{k}\right)$ represents the lookup table data points composed of historical observations or design values of discharge and tail water level.

To linearize the vibration zone avoidance constraint, i.e., Equation (21), this paper introduces auxiliary binary decision variables $\delta_{i,k,t}\in \left\{0,1\right\}$ and employs the big-M method to transform the original nonlinear product constraint into the following linear inequality form:

(37) $$\begin{array}{c} P_{i,t}^{\mathrm{hydro}}\leq P_{\min,i,k}^{\mathrm{hydro}}+M\cdot \delta_{i,k,t}\\ P_{i,t}^{\mathrm{hydro}}\geq P_{\max,i,k}^{\mathrm{hydro}}-M\cdot \left(1-\delta_{i,k,t}\right)\\ \delta_{i,k,t}\in \left\{0,1\right\} \end{array}$$

where $P_{i,t}^{\mathrm{hydro}}$ is the output of hydropower station i during period t, $\left[P_{\min,i,k}^{\mathrm{hydro}},P_{\max,i,k}^{\mathrm{hydro}}\right]$ are the upper and lower limits of the k-th vibration zone, $\delta_{i,k,t}$ indicates which side of the vibration zone the output is on, and M is a sufficiently large constant. This linearization method ensures that unit output does not fall into vibration zones, effectively avoiding operational risks caused by mechanical vibrations.

For the output fluctuation duration constraint, i.e., Equation (27), to ensure that hydropower unit output has minimum duration during increase and decrease processes and avoid frequent fluctuations, this paper proposes a trend consistency linearization method based on time windows. This method forces unit output to maintain unidirectional changes within specified time windows by introducing window-level binary variables. At each scheduling period t, a moving window of length $t_{e}$ is defined, and window-level trend variables $y_{i,t}\in \left\{0,1\right\}$ are introduced to represent the output change trend of unit i within window $\left[t-t_{e}+1,t\right]$, where $y_{i,t}=1$ indicates that unit output continuously increases within this window; $y_{i,t}=0$ indicates that unit output continuously decreases within this window. Let the power increment be:

(38) $$\Delta P_{i,k}^{\mathrm{hydro}}=P_{i,k}^{\mathrm{hydro}}-P_{i,k-1}^{\mathrm{hydro}},k=t-t_{e}+1,\ldots ,t$$

Then the consistency constraint for output fluctuation direction can be modeled as:

(39) $$\begin{array}{c} \Delta P_{i,k}^{\mathrm{hydro}}\geq -M\cdot \left(1-y_{i,t}\right),\forall k=t-t_{e}+1,\ldots ,t\\ \Delta P_{i,k}^{\mathrm{hydro}}\leq M\cdot y_{i,t},\forall k=t-t_{e}+1,\ldots ,t \end{array}$$

where M is a sufficiently large constant, typically taken as the maximum output change range of the unit.

To linearize the nonlinear function of head loss, i.e., Equation (30), this paper employs a sawtooth piecewise linear fitting method, dividing the quadratic form $H_{i,t}^{\mathrm{hydro},\mathrm{loss}}=a_{i}{\left(Q_{i,t}^{\mathrm{hydro}}\right)}^{2}+b_{i}$ into K linear intervals over the interval $\left[Q_{i}^{\min},Q_{i}^{\max}\right]$, with nodes recorded as $\left\{q_{i,0},q_{i,1},\ldots ,q_{i,K}\right\}$. In each k-th interval $\left[q_{i,k-1},q_{i,k}\right]$, a linear function is fitted:

(40) $$H_{i,t}^{\mathrm{hydro},\mathrm{loss}}\leq m_{i,k}\cdot Q_{i,t}^{\mathrm{hydro}}+c_{i,k}+M\left(1-\delta_{i,k,t}\right),\forall k=1,\ldots ,K$$

where $m_{i,k}$ is the slope of the k-th segment; $c_{i,k}$ is the intercept, fitted according to the quadratic function at interval endpoints; $\delta_{i,k,t}\in \left\{0,1\right\}$ is a binary decision variable indicating whether this segment is activated; M is a sufficiently large positive number ensuring that inactive segments do not affect the results.

The following mutual exclusion constraint is also added to ensure that only one segment is activated per period:

(41) $$\overset{K}{\underset{k=1}{\sum }}\delta_{i,k,t}=1$$

This paper employs a bivariate piecewise linear fitting method to handle unit dynamic characteristic constraints, i.e., Equation (31), dividing flow $Q_{i,t}^{\mathrm{hydro}}$ and head $H_{i,t}^{\mathrm{hydro}}$ into $N_{Q}$ and $N_{H}$ segments, respectively, constructing two-dimensional grid nodes $\left(q_{m},h_{n}\right)$, and defining the power value at each node as:

(42) $$p_{i,mn}=\eta_{i}\cdot \rho \cdot g\cdot q_{m}\cdot h_{n}$$

Continuous interpolation weight variables $\lambda_{i,mn,t}\in \left[0,1\right]$ are introduced to represent the contribution degree of node $\left(q_{m},h_{n}\right)$ to the output of unit i during period *t*:

(43) $$P_{i,t}^{\mathrm{hydro}}=\overset{N_{Q}}{\underset{m=1}{\sum }}\overset{N_{H}}{\underset{n=1}{\sum }}\lambda_{i,mn,t}\cdot p_{i,mn}$$

(44) $$Q_{i,t}^{\mathrm{hydro}}=\overset{N_{Q}}{\underset{m=1}{\sum }}\overset{N_{H}}{\underset{n=1}{\sum }}\lambda_{i,mn,t}\cdot q_{m}$$

(45) $$H_{i,t}^{\mathrm{hydro}}=\overset{N_{Q}}{\underset{m=1}{\sum }}\overset{N_{H}}{\underset{n=1}{\sum }}\lambda_{i,mn,t}\cdot h_{n}$$

(46) $$\overset{N_{Q}}{\underset{m=1}{\sum }}\overset{N_{H}}{\underset{n=1}{\sum }}\lambda_{i,mn,t}=1,\lambda_{i,mn,t}\geq 0$$

This method approximates the bivariate product relationship using linear interpolation, accurately reflecting the nonlinear characteristics of output variation with head and flow changes while ensuring model linearity. By adjusting segmentation precision (i.e., the values of $N_{Q}$ and $N_{H}$), flexible trade-offs can be made between accuracy and computational efficiency, applicable to hydropower output modeling under various scheduling scales.

### 3.2. Uncertainty in Scheduling Model

In the objective function, the PV curtailment cost term *F*_3_ introduces uncertainty in PV output. In constraint conditions, PV output constraint Equation (32), power balance constraint Equation (33), and spinning reserve constraint Equation (34) all consider uncertainty. This paper employs fuzzy mathematics methods to introduce fuzzy parameters and converts them to deterministic crisp equivalent forms under given confidence levels, thereby enhancing model feasibility in addressing PV and load uncertainties.

When considering PV output uncertainty, this paper models the predicted PV output $P_{j,t}^{\mathrm{pv},f}$ as triangular fuzzy parameters and performs crisp processing under confidence level α to obtain the equivalent confidence output lower bound:

(47) $${\hat{P}}_{j,t,\alpha }^{\mathrm{pv}}=\left(2-2\alpha \right)P_{j,t,2}^{\mathrm{pv}}+\left(2\alpha -1\right)P_{j,t,1}^{\mathrm{pv}}$$

Thus, the objective function *F*_3_ is extended to an uncertain form:

(48) $$F_{3}^{\mathrm{unc}}=\overset{T}{\underset{t=1}{\sum }}\overset{J}{\underset{j=1}{\sum }}\left({\hat{P}}_{j,t,\alpha }^{\mathrm{pv}}-P_{j,t}^{\mathrm{pv}}\right)$$

For PV output constraints, this paper converts them under confidence level α:

(49) $$\left\{\begin{array}{c} \left(2-2\alpha \right)P_{j,t,2}^{\mathrm{pv}}+\left(2\alpha -1\right)P_{j,t,1}^{\mathrm{pv}}\geq P_{j,t}^{\mathrm{pv}}\\ P_{j,t}^{\mathrm{pv}}\geq 0 \end{array}\right.$$

where $P_{j,t,1}^{\mathrm{pv}}$ and $P_{j,t,2}^{\mathrm{pv}}$ are the lower and upper membership endpoints of PV output prediction values, respectively.

For power balance constraints and spinning reserve constraints, this paper crisp-converts both under confidence level α:

(50) $$\begin{array}{l} \sum_{i=1}^{I}P_{i,t}^{\mathrm{hydro}}+\sum_{m=1}^{M}P_{m,t}^{\mathrm{fire}}+(2-2\alpha )\left(\sum_{j=1}^{J}P_{j,t,3}^{\mathrm{pv}}-\sum_{n=1}^{N}P_{n,t,2}^{\mathrm{load}}\right)\\ \;+(2-2\alpha )\left(\sum_{j=1}^{J}P_{j,t,4}^{\mathrm{pv}}-\sum_{n=1}^{N}P_{n,t,1}^{\mathrm{load}}\right)=0 \end{array}$$

(51) $$\begin{array}{l} -\sum_{i=1}^{I}P_{i,\max}^{\mathrm{hydro}}-\sum_{m=1}^{M}P_{m,\max}^{\mathrm{fire}}+(2-2\alpha )\left(\sum_{j=1}^{J}P_{j,t,3}^{\mathrm{load}}-\sum_{n=1}^{N}P_{n,t,2}^{\mathrm{pv}}\right)\\ \;+(2\alpha -1)\left(\sum_{j=1}^{J}P_{j,t,4}^{\mathrm{load}}-\sum_{n=1}^{N}P_{n,t,1}^{\mathrm{pv}}\right)\leq 0 \end{array}$$

## 4. Case Study

### 4.1. Tested System and Parameter Settings

**Figure 1:**
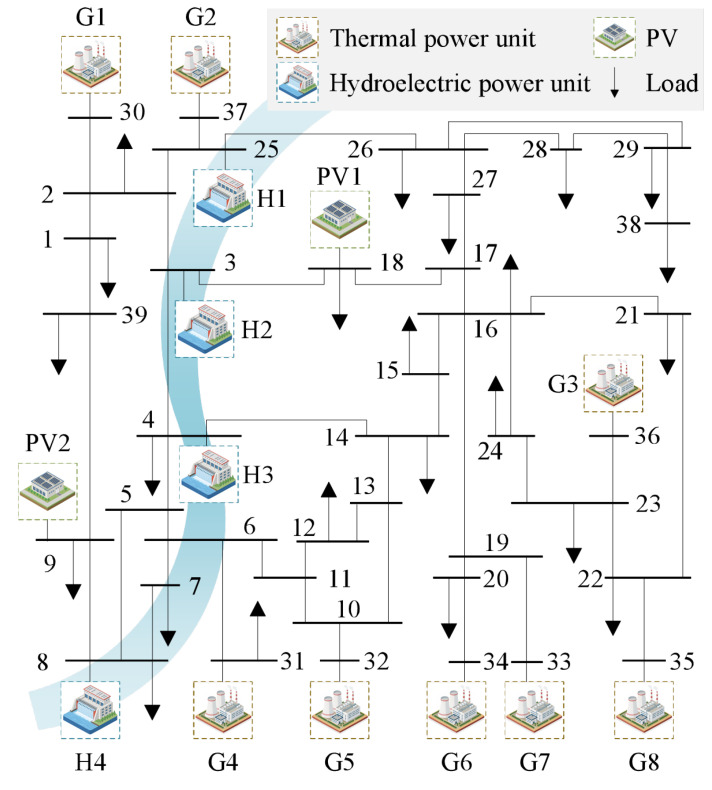
Topology diagram of modified IEEE −39 power system.

The modified IEEE −39 system is applied for the case study. It consists of eight thermal power units, four hydroelectric power units, and two PV plants, as illustrated in Figure 1. The detailed parameters of thermal and hydroelectric power units are listed in Table 1 and Table 2, respectively. For the PV generation, two plants are integrated into the system, with rated capacities of 1400 MW (PV1) and 600 MW (PV2), corresponding to a penetration rate of approximately 29% (ratio of all PVs’ capacity to the whole power generations’ capacity [31]).

**Figure 2:**
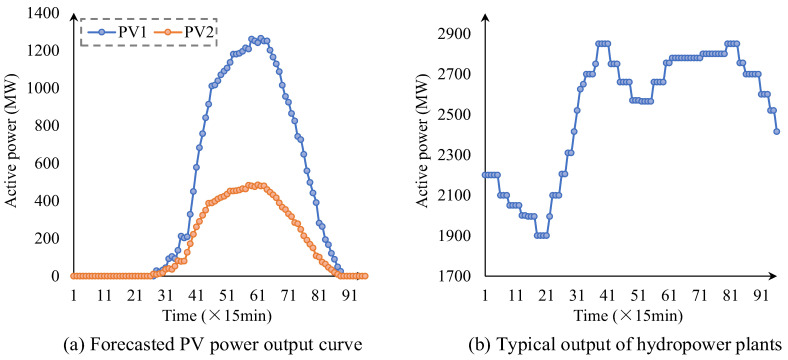
Forecasted PV power output and typical output of hydropower plants.

The forecasted power output curves of the two PV plants are illustrated in Figure 2a, showing the typical daily variation in solar power under different irradiation conditions. The generation schedule of the cascaded hydropower plants is presented in Figure 2b, exhibiting a clear time-varying pattern. Specifically, the output decreases during the early periods of the day, followed by a gradual increase. In the mid-day to evening hours, the hydropower output reaches relatively higher levels and remains stable.

### 4.2. Analysis of Scheduling Results

To characterize the impact of uncertainty on scheduling results, this study introduces different confidence levels α. The confidence level α represents the probability threshold at which the scheduling solution remains feasible under uncertainties. Higher α values indicate conservative strategies requiring feasibility in more scenarios, suitable for systems with strict reliability requirements, while lower α values allow greater risk tolerance to maximize renewable accommodation. In practice, α should be selected considering system reliability standards, historical forecast error distributions, and economic trade-offs between curtailment and generation costs. The confidence level range (α= 0.98, 0.96, 0.94, 0.92, 0.90, 0.88) reflects typical reliability standards in power system scheduling.

**Figure 3:**
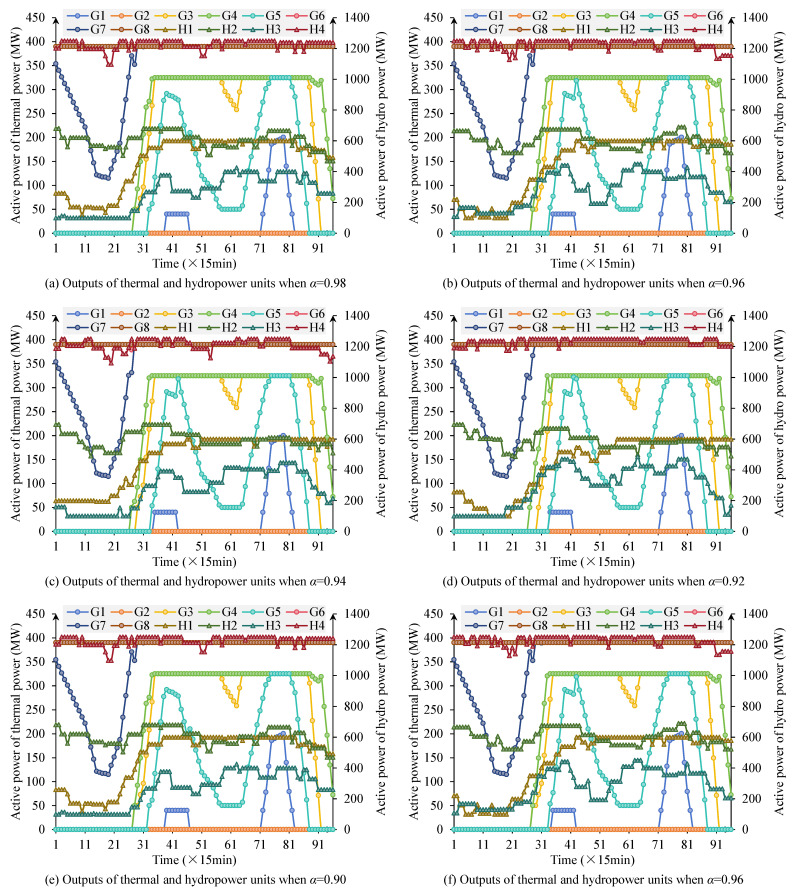
Scheduling results of thermal and hydropower units under different confidence levels *α*.

Figure 3 shows the output variation trends of thermal and hydropower units under different confidence levels. When the confidence level is high, the system has strong requirements for preventing stochastic risks. To satisfy strict feasibility requirements under uncertainty, the optimizer adopts conservative strategies by increasing thermal reserves and reducing dependence on variable renewables, representing risk-averse operation suitable for systems with critical reliability requirements. At this stage, thermal units maintain relatively high output levels overall, accompanied by obvious start-stop adjustments to ensure system reliability. The regulation capability of hydropower units is also fully utilized, with output showing strong following characteristics to compensate for PV output fluctuations. During this phase, renewable energy accommodation is somewhat suppressed, PV utilization is insufficient, and curtailment phenomena are prominent. As the confidence level gradually decreases, the scheduling strategy’s tolerance for uncertainty risks increases. Relaxed feasibility constraints enable the optimizer to exploit statistical expectations of renewable output rather than worst-case scenarios, reducing conservative reserve requirements. This manifests as a gradual decline in thermal unit operating frequency and average output, hydropower unit output becoming more stable, and PV and other renewable energy sources receiving more adequate accommodation in the system. Particularly at α=0.88, PV output is utilized to a significant extent, and the overall system operational mode shifts from “conservative” to “economic”. This demonstrates that systems with abundant flexibility resources can adopt lower confidence levels to maximize economic and environmental benefits while maintaining acceptable reliability.

Table 3 lists the three types of objective function values under different confidence levels, further validating the scheduling trends revealed in Figure 3. Specifically, thermal power operating cost *F*_1_ gradually decreases as the confidence level decreases, from 1.584 × 10^6^$ at α=0.98 to 1.538 × 10^6^$ at α=0.88, indicating that thermal unit output decreases under lower confidence levels and system operating costs are effectively controlled. The hydropower deviation index *F*_2_ gradually increases as the confidence level decreases, from 215.4 MW to 223.5 MW, suggesting that under low confidence levels, the difference between hydropower units and expected output expands, with the system trading precise hydropower scheduling constraints for better economic performance and renewable energy accommodation. The PV curtailment index *F*_3_ shows a decreasing trend as the confidence level decreases, from 268.9 MW to 253.2 MW, indicating that under low confidence levels, PV output can be more fully integrated into the grid, and curtailment phenomena are significantly improved. Overall, high confidence levels correspond to high reliability but higher costs and severe curtailment, while low confidence levels achieve reduced operating costs and improved PV accommodation at the expense of some hydropower scheduling precision.

The regulation coefficient β dynamically limits thermal power output to prioritize renewable energy accommodation. Larger β values provide thermal units more operational freedom, suitable for early-stage renewable integration, while smaller β values enforce renewable priority for high-penetration scenarios. In practice, β should be selected based on renewable policy targets, thermal unit economic operation ranges, system flexibility requirements, and seasonal renewable patterns, with potential for real-time adjustment. This paper sets β=0.90, 0.87, 0.84, 0.81, for comparative analysis.

**Figure 4:**
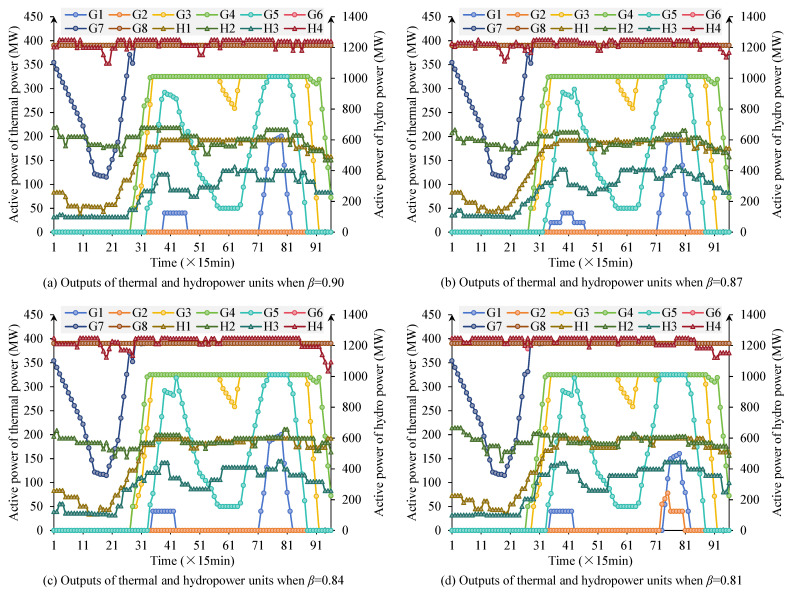
Scheduling results of thermal and hydropower units under different regulation coefficients *β*.

Figure 4 shows the output scheduling curves of thermal and hydropower units under different β values. From the results, it can be seen that as β decreases, thermal unit output is gradually suppressed, the number of start-stop operations of some units decreases, and overall operating levels decline. Smaller β values impose tighter constraints on thermal generation, effectively forcing the system to prioritize renewable accommodation. When thermal power is constrained, hydropower must undertake more load-following and ramping tasks to maintain balance. Correspondingly, the regulation role of hydropower units is further enhanced, with output undertaking more load-following tasks and demonstrating strong ramping capabilities. This trend indicates that smaller β values can effectively suppress excessive thermal power output, making room for renewable energy accommodation. Meanwhile, PV output receives more grid utilization, and curtailment phenomena are alleviated. From an engineering perspective, β serves as a policy instrument for balancing renewable integration targets with system stability requirements and could be dynamically adjusted based on grid conditions and operational priorities. Overall, the smaller the β is, the more the system operating mode tends toward “renewable energy priority”; while the larger the β is, the more system operation relies on thermal power stability.

Table 4 quantitatively presents the three types of objective function values under different β values. From the results, it can be seen that thermal power operating cost *F*_1_ continuously decreases as β decreases, from 1.584 × 10^6^$ at β=0.90 to 1.518 × 10^6^$ at β=0.81. This indicates that by tightening thermal power output upper limits, the system reduces its dependence on thermal units, effectively controlling operating costs. The hydropower deviation index *F*_2_ gradually increases as β decreases, from 214.2 MW to 224.8 MW, reflecting that under thermal power limitations, hydropower undertakes more load regulation tasks, with increased deviations from expected output. The PV curtailment index *F*_3_ significantly decreases as β decreases, from 268.9 MW to 253.8 MW, indicating that after thermal power output is limited, PV grid output is significantly improved, and curtailment phenomena are alleviated.

### 4.3. Scheduling Under Different PV Penetration Levels

**Figure 5:**
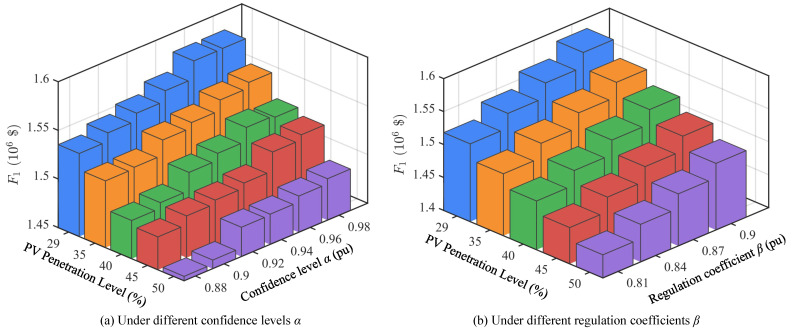
Values of objective function *F*_1_ under different PV penetration levels.

**Figure 6:**
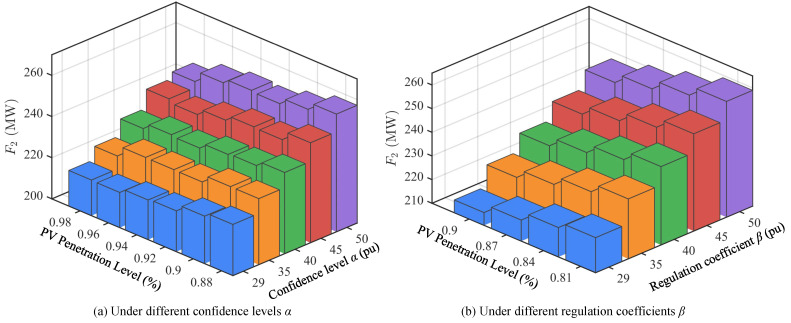
Values of objective function *F*_2_ under different PV penetration levels.

**Figure 7:**
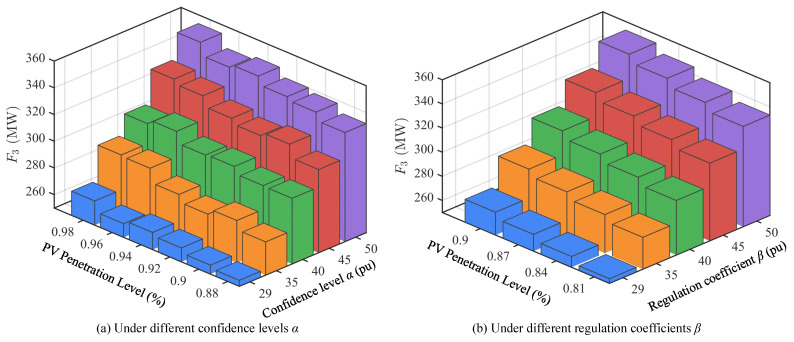
Values of objective function *F*_3_ under different PV penetration levels.

To further evaluate the impact of large-scale PV integration on power system optimal scheduling, this paper conducts comparative analysis of scheduling results under different PV penetration levels (29–50%), as shown in Figure 5, Figure 6 and Figure 7. The research focuses on three objective functions: thermal power operating cost *F*_1_, hydropower output deviation index *F*_2_, and PV curtailment index *F*_3_, and examines the impact of different confidence levels α and regulation coefficients β on scheduling results.

Figure 5 shows the values of objective function *F*_1_ under different PV penetration levels. It can be seen that as PV penetration gradually increases, system dependence on thermal units decreases significantly, resulting in an overall declining trend in operating costs. For example, under conditions of α=0.98 and β=0.90, *F*_1_ decreases from approximately 1.584 × 10^6^$ at 29% penetration to approximately 1.501 × 10^6^$ at 50% penetration. This trend remains consistent under different confidence levels and regulation coefficients, indicating that increasing PV share can effectively reduce system economic operating costs, but also increases system adaptation requirements for renewable energy fluctuations.

Figure 6 shows the values of objective function *F*_2_ under different PV penetration levels. Contrary to *F*_1_, *F*_2_ gradually increases with increasing PV penetration, rising from approximately 215 MW at 29% to over 250 MW at 50%. This indicates that under high-proportion PV integration, hydropower units undertake more regulation tasks to balance PV output fluctuations and uncertainties. Therefore, hydropower unit scheduling flexibility requirements continue to strengthen, and their operational deviations expand accordingly. This result highlights the important role of hydropower as the main flexible regulation resource under high penetration scenarios.

Figure 7 shows the values of objective function *F*_3_ under different PV penetration levels. From the changes in PV curtailment index *F*_3_, as penetration increases, curtailment shows a gradual upward trend. At 29% PV penetration, curtailment is relatively low, while at 50% penetration, *F*_3_ has exceeded 330 MW. This indicates that under insufficient system regulation capability, high-proportion PV integration inevitably brings higher curtailment risks. However, reasonable settings of regulation coefficient β and confidence level α can alleviate curtailment problems to some extent, enabling more effective utilization of renewable energy sources.

In summary, scheduling results under different PV penetration levels show significant differences. As PV penetration increases, thermal power operating costs continue to decrease, reflecting the gradual weakening of system dependence on thermal units; meanwhile, hydropower deviation indices show an increasing trend, indicating that hydropower units undertake more regulation tasks; PV curtailment indices also gradually increase with penetration, reflecting more obvious PV output limitations under high penetration conditions.

### 4.4. Error Analysis of Nonlinear Constraint Linearization

In this section, the accuracy of handling nonlinear constraints in the scheduling model is evaluated. The analysis focuses on the linearization of the water level–storage and tail water level–discharge relationships, the effectiveness of vibration zone constraints, the impact of output fluctuation duration constraints, and the approximation of head loss and power surface functions.

The operating ranges of typical cascade hydropower stations are selected as test conditions, where the upstream water level varies within the 800–880 m range, corresponding to storage capacities of 1.2 × 10^8^–3.6 × 10^8^ m^3^; unit discharge ranges from 300 to 1000 m^3^/s, corresponding to tail water levels between 120 and 138 m. Based on data from this operating range, the linearization accuracy of water level–storage and tail water level–discharge relationships is analyzed, with results shown in Table 5 and Table 6. From the comparison results, it can be seen that the piecewise linear interpolation method can well approximate the original nonlinear curves. For the water level–storage relationship, errors between fitted values and actual values are small, with overall relative errors consistently controlled within 1%, indicating that storage estimation can maintain high reliability. In the tail water level–discharge relationship, piecewise linear approximation also shows good fitting performance, with relative errors all below 0.5%, accurately reflecting the variation pattern of tail water levels with discharge. Comprehensively, the piecewise linearization method significantly reduces the nonlinear complexity of the model while ensuring low error levels, providing efficient and accurate mathematical expressions for subsequent scheduling optimization. This precision level fully meets the requirements of practical scheduling applications.

This paper adopts linearized vibration zone avoidance constraints aimed at preventing hydropower units from entering mechanical vibration zones during scheduling, preventing equipment wear or operational risks caused by long-term operation in unstable conditions. Case study results show that in all operating scenarios set in this paper, each unit does not enter its respective vibration zone throughout the entire scheduling period, indicating that the constraints effectively function. Taking Figure 3a as an example, H1’s output always remains above 150 MW or far above the upper limit, avoiding the (90–150 MW) vibration zone; H2’s output is mostly above 500 MW, not entering the (105–175 MW) unstable interval; H3’s minimum output maintains at 100 MW, above the (60–100 MW) boundary; H4’s output basically maintains at 1150–1250 MW, avoiding the (190–320 MW) interval. This shows that the proposed vibration zone avoidance constraints achieve the expected goal during the optimization scheduling process, namely using auxiliary variables and the big-M method to logically separate unit output from vibration zone intervals, making the feasible solution space automatically avoid unsafe intervals. The results not only ensure the safety and stability of operating curves but also verify the feasibility and practical value of this method in complex scheduling models.

Next, the impact of output fluctuation duration constraints (minimum duration time) is analyzed. This paper introduces output fluctuation duration constraints in the scheduling model, stipulating that units must maintain a certain minimum duration time during power increase or decrease processes to avoid short-term frequent fluctuations. To verify the effectiveness of this constraint, this paper sets two scenarios for comparison: one is “without considering output fluctuation constraints,” and the other is “considering minimum duration constraints (4 periods),” with results shown in Table 7. It can be seen that without considering constraints, hydropower unit output curves show multiple rapid increase-decrease switches, with cumulative fluctuation counts reaching 14 times, and some continuous power increase processes lasting only 1 period. After considering minimum duration constraints, unit fluctuation counts decrease to 6 times, with single increase-decrease process durations all exceeding 4 periods, making curve changes smoother. Further comparison of system operating indices reveals that although introducing this constraint slightly reduces hydropower-thermal coordination, leading to approximately 1.1% increase in overall operating costs, it effectively avoids frequent hydropower regulation and improves system stability.

**Figure 8:**
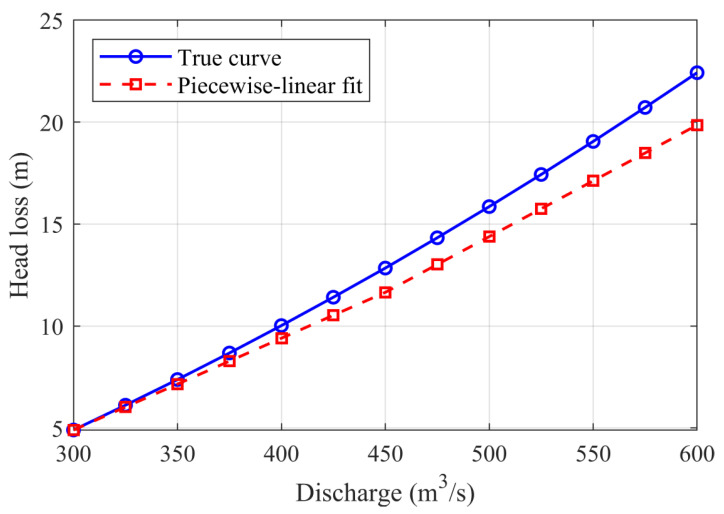
Head loss versus discharge: true values and piecewise-linear fitting.

To ensure modeling accuracy while reducing the nonlinear complexity of optimization problems, this paper adopts piecewise linear and piecewise bilinear approximations for the head loss function $h_{l}(Q)$ and unit dynamic characteristics P(H,Q) (power-head-discharge bivariate relationship), respectively. First, within the discharge range $Q\in [300,600]\;m^{3}/s$, two-segment piecewise linear functions are constructed using endpoint interpolation (segment points 300, 450, 600), and fitting errors are evaluated at 25 m^3^/s fine-grain sampling, with results shown in Figure 8. It can be seen that piecewise linear fitting can well approximate the original head loss curve, with mean absolute error (MAE) of approximately 0.021 m, maximum absolute error of approximately 0.042 m, and relative error peak not exceeding 0.45%, indicating that this approximation is sufficient to replace the original nonlinear relationship at the scheduling scale.

**Figure 9:**
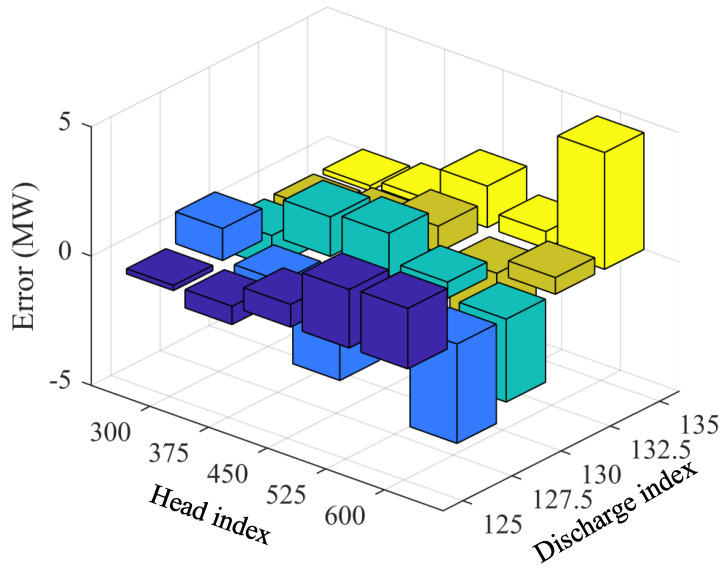
Errors between true and bilinear fitted power surfaces under different head and discharge conditions.

For unit dynamic characteristics P(H,Q), within the head range H∈[125,135] m and discharge range $Q\in [300,600]\;m^{3}/s$, segment points H = 125, 127.5, 130, 132.5, 135 and Q = 300, 375, 450, 525, 600 are selected to construct a two-dimensional grid for bilinear interpolation approximation of the power surface, with absolute errors between fitted results and actual values shown in Figure 9. It can be seen that the maximum absolute error is approximately 2.078 MW, with relative error peak not exceeding 0.74%. Therefore, using a small number of segments can effectively linearize the bivariate coupling P(H,Q) while ensuring high accuracy. The segmentation scheme adopted in this study achieves a good balance between engineering computability and approximation accuracy, providing a stable and reliable linear approximation model for scheduling optimization.

### 4.5. Comparison with Other Method

As a reference method, we construct a full nonlinear mixed-integer benchmark (MINLP) that preserves all hydropower physics without linearization. The water level–storage and tailwater–discharge relations are modeled by cubic-spline interpolation; head-loss is kept in its quadratic form; and the turbine power–head–discharge coupling is represented by the original bilinear/quadratic equations. Vibration-zone avoidance and minimum output-duration constraints are imposed with binary variables (big-M) and logical linking constraints (the same logic as in the proposed model, but without linear surrogates for the physics). The day-ahead horizon, discretization, initial/terminal levels, PV/load scenarios, and all operational bounds are identical to the proposed method to ensure fairness. The MINLP is solved by a state-of-the-art nonlinear branch-and-bound solver with relative/absolute optimality tolerances of 10^−4^/10^−6^, feasibility tolerance 10^−6^, and a wall-clock limit of 3600 s; warm starts are disabled to avoid bias.

We compared the (i) objective value and relative optimality gap, (ii) CPU time, and (iii) constraint violations of the proposed method and the compared method. The results are shown in Table 8, Table 9 and Table 10. Based on these results, the Proposed method demonstrates clear advantages over the Compared method in both computational efficiency and practical accuracy. As shown in Table 8, the Proposed method achieves nearly identical objective value (1.584 × 10^6^$ vs. 1.592 × 10^6^$) while reaching a proven optimal solution with zero optimality gap, whereas the Compared method retains a 1.2% residual gap after reaching the time limit. Table 9 further indicates that the Proposed method converges within 182 s—over 20 times faster than the Compared method, which requires the full 3600 s time limit without full convergence. Regarding model fidelity (Table 10), the Proposed method maintains small approximation errors in the hydropower physics—storage, tailwater level, and head-loss MAEs remain below 1% of their respective ranges—and negligible power-balance violations, confirming that linearization does not significantly compromise accuracy. Overall, these results verify that the Proposed method achieves a favorable balance between modeling precision and computational tractability, making it more suitable for large-scale, time-sensitive power-system scheduling applications.

## 5. Conclusions

This paper develops an optimization-based scheduling method for coordinating hydropower, thermal power, and PV generation under uncertainty and nonlinearity. A detailed hydropower model is formulated to capture water balance, reservoir dynamics, and nonlinear head–discharge–power characteristics. To enhance computational tractability, nonlinear constraints are approximated through piecewise and bilinear linearization, while uncertainty is represented using fuzzy parameters with confidence-level reformulation. Case studies on the modified IEEE −39 system verify that the proposed method achieves effective coordination among multi-type sources. Quantitatively, the results demonstrate the framework’s effectiveness across varying conditions: operating costs vary by 2.9% across confidence levels (α = 0.98 to 0.88), renewable accommodation improves with adjusted thermal constraints (β = 0.9 to 0.81), and the method remains computationally tractable for PV penetration levels up to 50%. These results demonstrate the practicality of the proposed scheduling framework and its potential to support reliable and economical operation of future low-carbon power systems. However, the current study is limited by the assumption of static hydrological and load conditions, and further research is required to validate the proposed framework under time-varying inflow patterns and network-level constraints. Future work should focus on validating the approach on larger-scale realistic systems with diverse renewable portfolios and complex transmission constraints to establish scalability and generalizability. Additionally, developing real-time adaptive strategies capable of responding to forecast updates and system disturbances represents a critical priority, where machine learning techniques could enhance prediction accuracy and decision-making efficiency under the inherent uncertainties of renewable energy integration.

## Figures and Tables

**Table 1:** Thermal power unit parameters.

Parameter	G1	G2	G3	G4	G5	G6	G7	G8
Rated power (MW)	200	200	250	250	250	300	300	300
Minimum technical output (MW)	40	40	50	50	50	60	60	60
Startup cost ($)	600	700	800	800	900	1400	1500	1500
Minimum on/off time (h)	2	2	3	3	3	4	4	4
Ramp rate (MW/min)	4.5	4.9	5.4	5.9	6	7.5	8	8.8
Electricity purchase cost coefficient 1 ($/MW·h)	54.82	55.64	43.68	43.08	44.51	37.12	37.36	35.96
Electricity purchase cost coefficient 2 ($/h)	105.18	104.25	116.02	117.41	115.44	122.62	122.27	123.57
Up reserve cost coefficient ($/MW·h)	11.76	12.82	15.81	15.37	14.58	17.58	17.21	18.01
Down reserve cost coefficient ($/MW·h)	10.90	10.92	13.32	12.11	11.73	15.22	14.66	14.22
Droop coefficient	0.042	0.042	0.042	0.042	0.042	0.043	0.043	0.043
Governor proportional gain	6	10	17	15	12	16	19	19
Governor integral gain	2.4	2.4	2.5	2.5	2.6	2.4	2.6	2.7

**Table 2:** Main characteristic parameters of cascade hydropower plant.

Parameter	Single Unit Capacity (MW)	Vibration Zone (MW)	Unit Start/Stop Duration (h)	Stable Output Duration (h)	Stall Time (h)
H1	600	(90, 150)	2	1	1
H2	700	(105, 175)	2	1	1
H3	400	(60, 100)	2	1	1
H4	1250	(190, 320)	2	1	1

**Table 3:** Objective function values under different confidence levels *α*.

Objective	*F*_1_ ($)	*F*_2_ (MW)	*F*_3_ (MW)
*α* = 0.98	1.584 × 10^6^	215.4	268.9
*α* = 0.96	1.572 × 10^6^	216.7	264.5
*α* = 0.94	1.566 × 10^6^	218.7	261.3
*α* = 0.92	1.553 × 10^6^	220.1	258.4
*α* = 0.90	1.544 × 10^6^	221.3	255.9
*α* = 0.88	1.538 × 10^6^	223.5	253.2

**Table 4:** Objective function values under different regulation coefficients *β*.

Objective	*F*_1_ ($)	*F*_2_ (MW)	*F*_3_ (MW)
*β* = 0.9	1.584 × 10^6^	214.2	268.9
*β* = 0.87	1.562 × 10^6^	217.5	262.5
*β* = 0.84	1.543 × 10^6^	219.9	257.4
*β* = 0.81	1.518 × 10^6^	224.8	253.8

**Table 5:** Water level–storage relationship and fitting accuracy.

Water Level (m)	Storage (10^8^ m^3^)	Fitted Value (10^8^ m^3^)	Error (10^8^ m^3^)	Relative Error (%)
800	1.20	1.20	0.00	0.0
820	1.95	1.93	0.02	1.0
840	2.55	2.53	0.02	0.8
860	3.15	3.13	0.02	0.6
880	3.60	3.59	0.01	0.3

**Table 6:** Tail water level–discharge relationship and fitting accuracy.

Discharge (m^3^/s)	Tail Water Level (m)	Fitted Value (m)	Error (m)	Relative Error (%)
300	120.0	120.0	0.00	0.0
500	125.5	125.3	0.20	0.2
700	130.2	130.5	0.30	0.2
900	134.0	134.3	0.30	0.2
1000	138.0	138.2	0.20	0.1

**Table 7:** Comparison of scheduling with and without output fluctuation duration constraint.

Scenario	Number of Fluctuations	Minimum Duration (×15 min)	Average Duration (×15 min)	PV Curtailment (MW)	Operating Cost (×10^6^$)
Without constraint	14	1	2.1	268.5	1.546
With constraint	6	3	4.5	259.1	1.563

**Table 8:** Objective value and relative optimality gap.

Method	Objective (×10^6^$)	Relative Optimality Gap (%)
Proposed method	1.584	0.00
Compared method	1.592	1.20

**Table 9:** Computational efficiency.

Method	CPU Time (s)	Nodes (×10^3^)
Proposed method	182	34
Compared method	3600	118

**Table 10:** Constraint fidelity and residuals.

Method	Storage MAE (10^8^ m^3^)	Tailwater MAE (m)	Head-Loss MAE (m)	Power Surface MAE (MW)	Power-Balance Max Viol. (MW)
Proposed method	0.018	0.18	0.025	1.2	<1 × 10^−4^
Compared method	0.000	0.001	0.000	0.02	<1 × 10^−6^

## Data Availability

The data presented in this study are available on request from the corresponding author due to privacy restrictions.

## References

[1] Liang N., Fang L., Xu H., Zheng F., Miao Y. (2024). Bi-level optimal dispatching of power system based on demand response considering nodal carbon intensity. Autom. Electr. Power Syst..

[2] Hou T., Fang R., Wang Z., Hou J., Fan X., Huang B. (2024). Optimal scheduling and compensation cost allocation mechanism to enhance the initiative of source–load multi-element peak regulation. High Volt. Eng..

[3] Yang X., Liu X., Guo Q., Sun Y., Yan Q., Li H. (2023). Coordinated planning of energy storage and flexible retrofit of thermal power units considering ancillary service income. Power Syst. Technol..

[4] Cao P., Deng C., Zhang X., Zhang Y., Dong Y. (2025). Real-time coordinated optimal scheduling strategy for thermal power units with adaptive time division and variable objective. Power Syst. Technol..

[5] Yuan M., Cai J., Chen Y., Lou J., Mao W., Yan G., Liu Y., Zhang W. (2024). Ahead dispatch of asynchronous interconnected sending end power grid considering HVDC frequency support. Power Syst. Technol..

[6] Liu X., Zu L., Li X. (2023). Day-ahead and intra-day economic dispatch of electricity–hydrogen integrated energy system with virtual energy storage. IEEE Access.

[7] Zhong L., Zhang J., Chung C.Y., Gong Y., Guan L. (2022). Dynamic-decision-based real-time dispatch for reducing constraint violations. J. Mod. Power Syst. Clean Energy.

[8] Chu Z., Zhang N., Teng F. (2021). Frequency-constrained resilient scheduling of microgrid: A distributionally robust approach. IEEE Trans. Smart Grid.

[9] Yang L., Li Z., Xu Y. (2022). Frequency-constrained scheduling under multiple uncertainties via data-driven distributionally robust chance-constrained approach. IEEE Trans. Sustain. Energy.

[10] Ye L., Zhang B., Guo K., Pei M., Xia X., Xie H. (2025). Optimal collaborative dispatching of wind–thermal combined power generation system in the day-ahead and intra-day stages. CSEE J. Power Energy Syst..

[11] Yang L., Xu Y., Zhou J., Sun H. (2022). Distributionally robust frequency constrained scheduling for an integrated electricity–gas system. IEEE Trans. Smart Grid.

[12] Prakash V., Sharma K.C., Bhakar R. (2017). Frequency response constrained modified interval scheduling under wind uncertainty. IEEE Trans. Sustain. Energy.

[13] Tan H., Chen J., Wang Q., Weng H., Li Z., Mohamed M.A. (2025). Day-ahead wind power admissibility assessment of power systems considering frequency constraints. IEEE Trans. Ind. Inf..

[14] Jia Y., Lin M., Dong Z. (2020). Research on optimal operation of hydro–photovoltaic complementarity in Longyangxia hydropower station. Water Resour. Power.

[15] Zhu Y., Chen S., Ma G., Han X., Wang L. (2020). Short-term complementary operation of hydro–photovoltaic integrated system considering power generation and output fluctuation. Trans. China Electrotech. Soc..

[16] Song K. (2021). Optimal scheduling of hydro–photovoltaic complementary generation system based on scene feature clustering. Water Resour. Power.

[17] Ming B., Liu P., Guo S.L. (2019). Hydropower reservoir reoperation to adapt to large-scale photovoltaic power generation. Energy.

[18] Luo B., Miao S., Qiu Y., Gao L., Chen G., Wang L. (2021). Operation safety analysis method for cascaded hydro–photovoltaic complementary generation system and its application. Sichuan Electr. Power Technol..

[19] Hu L., Li Y., Liu P., Wang Y., Ma C., Huang Q. (2021). Long-term optimal operation of hydro–solar hybrid energy systems nested with short-term energy curtailment risk. J. Hydraul. Eng..

[20] Wang L., Chen G., Miao S., Ding L., Han X., Wang X. (2020). A short-term optimal scheduling model of cascaded hydro–photovoltaic hybrid power generation system. Water Power.

[21] Han J., Chen Z. (2024). An inertial control method for large-scale wind farm based on hierarchical distributed hybrid deep-reinforcement learning. J. Clean. Prod..

[22] Han J., Lyu W., Song H., Qu Y., Wu Z., Zhang X., Li Y., Chen Z. (2023). Optimization of communication network for distributed control of wind farm equipped with energy storages. IEEE Trans. Sustain. Energy.

[23] Wang B., Yang D., Cai G. (2020). Dynamic frequency constraint unit commitment in large-scale wind power grid connection. Power Syst. Technol..

[24] Yuan Y., Zhang Y., Wang J., Liu Z., Chen Z. (2023). Enhanced frequency-constrained unit commitment considering variable-droop frequency control from converter-based generator. IEEE Trans. Power Syst..

[25] Li J., Qiao Y., Lu Z., Ma W., Cao X., Sun R. (2024). Integrated frequency-constrained scheduling considering coordination of frequency regulation capabilities from multi-source converters. J. Mod. Power Syst. Clean Energy.

[26] Wang L., Fan H., Liang J., Xu L., Li T., Luo P., Hu B., Xie K. (2024). Multi-area frequency-constrained unit commitment for power systems with high penetration of renewable energy sources and induction machine load. J. Mod. Power Syst. Clean Energy.

[27] Jiang B., Guo C., Chen Z. (2024). Modeling the coupling of rotor speed, primary frequency reserve, and virtual inertia of wind turbines in frequency-constrained look-ahead dispatch. IEEE Trans. Sustain. Energy.

[28] Wang Z., Wang Z., Liu M., Li G., Qi X., Miao S. (2021). Unit commitment model for high-proportion wind power system considering dynamic frequency response constraints. High Volt. Eng..

[29] Han J., Zhang D., Xie L., Luo C., Jia B., Li Q., Chen Z. (2025). Dispatch strategy for power system with high wind power penetration considering secondary frequency drop. IEEE Trans. Power Syst..

[30] Chen Y., Zhang Z., Chen H., Zheng H. (2022). Robust optimization based coordinated network and source planning considering uncertainty of renewable energy and load. IEEE Trans. Sustain. Energy.

[31] Han J., Zhang D., Jia B., Xie L., Wan W., Tan J., Chen Z. (2025). Power loss minimization-oriented reactive power control for wind farm equipped with distributed energy storages using clustering-based data-driven method. Energy.

